# Effects of Age on Cervical Vestibular Evoked Myogenic Potentials and Ocular Vestibular Evoked Myogenic Potentials Using 750 Hz Tone Burst Stimuli among Healthy Adults

**DOI:** 10.21315/mjms2022.29.4.6

**Published:** 2022-08-29

**Authors:** Sharifah Zainon Sayed, Nor Haniza Abdul Wahat

**Affiliations:** Audiology Program, Centre for Rehabilitation and Special Needs Studies, Faculty of Health Sciences, Universiti Kebangsaan Malaysia, Kuala Lumpur, Malaysia

**Keywords:** servical vestibular evoked myogenic potential, ocular vestibular evoked myogenic potential, vestibular function testing, age, gender

## Abstract

**Background:**

The vestibular evoked myogenic potentials (VEMPs) response characteristics depend on age, stimulus and individual anatomical differences. Therefore, normative data are required for accurate VEMPs interpretations. This cross-sectional study investigates VEMPs age-related changes among healthy adults using 750 Hz short alternating tone burst (TB) stimuli.

**Methods:**

Fifty adults aged between 23 years old and 76 years old with a mean of 51.56 (SD = 16.44) years old underwent air-conducted (AC) cervical VEMP (cVEMP) and head taps ocular VEMPs (oVEMPs) testing.

**Results:**

The cVEMPs and oVEMPs response rates reduced significantly at the age of 50-year-old and above. No significant age trends were observed for both cVEMPs and oVEMPs latencies and asymmetry ratios. However, amplitude reduced with increasing age for both cVEMPs, *P* < 0.001 and oVEMPs, *P* = 0.01. No significant differences in cVEMPs and oVEMPs latencies, amplitude or asymmetry ratios were identified between gender.

**Conclusion:**

To the best of our knowledge, this is the first published normative data for cVEMPs and oVEMPS in Malaysia and Southeast Asia, obtained among healthy adults aged between 23 years old and 76 years old. Health professionals in the region can use these findings as VEMPs normative references in their clinical settings.

## Introduction

Vestibular evoked myogenic potentials (VEMPs) are electrophysiological tests used widely to diagnose otolithic function and its afferent pathways. The saccular and utricular functions are evaluated by two VEMPs’ variants, namely cervical VEMPs (cVEMPs) and ocular VEMPs (oVEMPs). The cVEMPs are vestibulo-collic reflexes originating from the saccule and transmit through the inferior vestibular nerve (1–5). The saccular afferent pathways project to the vestibular nucleus in the brainstem and later descend to the ipsilateral sternocleidomastoid (SCM) muscles through the medial vestibulospinal tract (6, 7). The oVEMPs represent the vestibulo-ocular reflex from the utricle and superior vestibular nerve (1–5). The utricular afferent pathways project to the brainstem vestibular nuclei and oculomotor nuclei and later ascend to excite the contralateral inferior oblique muscle of the eye via the medial longitudinal fasciculus in the brainstem (6, 7).

VEMPs parameters, i.e. the latency, amplitude and asymmetry ratios, are mainly used to diagnose peripheral vestibular lesions such as vestibular neuritis, Meniere’s disease and superior canal dehiscence related to injuries involving the otolith organs (8–10). However, many studies found that both cVEMPs and oVEMPs parameters changed with increasing age, which could influence the diagnosis of vestibular dysfunction, especially in the elderly population. cVEMPs amplitude decreased with increasing age (11–13), while p13 prolonged in the elderly than the younger subjects (12, 13). For oVEMPs, earlier studies reported similar prolongation for latencies (11) and reduced amplitudes (11, 14) with increasing age.

VEMPs are evoked by short, intense auditory stimuli (i.e. tone bursts [TBs] or clicks), bone-conducted (BC) vibration, forehead taps and galvanic stimulation (3, 4). In the clinical setting, air-conducted (AC) stimuli and forehead taps are most commonly used for cVEMPs and oVEMPs. AC stimulus can elicit saccular responses robustly for cVEMPs, except in the presence of conductive losses (15–17). For oVEMPs, the forehead tap is usually used as it produces the the most prominent and optimal responses than the AC stimuli or BC stimuli (15). Although most studies used 500 Hz TB AC stimuli for cVEMPs and head taps stimuli for oVEMPs, 750 Hz TB AC stimuli might be ideal than 500 Hz TB AC stimuli especially in the older populations (18). The otolithic organs’ tuning curve might not be as sharp as the auditory tuning curve, with younger subjects having the highest peak amplitude at 500 Hz (18). In contrast, the highest amplitude was observed at 750 Hz or 1,000 Hz (18) for the older population. Therefore, 750 Hz TB AC stimuli may be ideal for elderly subjects. An earlier study also reported that cVEMPs elicited using 750 Hz TB AC stimuli had better sensitivity and specificity in detecting peripheral vestibular disorders than using 500 Hz or 1,000 Hz stimuli (19). The 750 Hz TB AC is considered a safer stimulus than 500 Hz TB AC due to its shorter duration and lower sound exposure level, thus reducing the risk of acoustic trauma on patients during testing (20, 21).

While ageing could alter VEMPs responses, other factors such as ethnicity, genetic and geometric anatomical differences might also influence VEMPs generations (11). The black ethnicity had shorter oVEMPs latencies than the whites and males had longer cVEMPs p13 latencies than females (11). Therefore, it is crucial to establish a set of VEMPs local reference data of specific demographic backgrounds to help clinicians diagnose vestibular disorders. This study aims to identify possible changes in response rates, latencies, amplitudes and asymmetry ratios of AC cVEMPs and forehead vibrations oVEMPs with increasing age among healthy local adults tested using 750 Hz TB stimuli.

## Methods

### Subjects

This cross-sectional study was conducted at the Vestibular Laboratory, Universiti Kebangsaan Malaysia (UKM) Audiology Programme between July 2018 and August 2019. Subjects selected were volunteers from UKM, Pusat Aktiviti Warga Emas (PAWE) Taman Wahyu and Pusat Aktiviti Warga Emas (PAWE) Seputeh, Kuala Lumpur with no history of otological or neurological disorders. Exclusion was made for subjects with spontaneous or gaze-evoked nystagmus, abnormal head impulse responses, or any signs or symptoms of dizziness or vertigo. The study was performed under the approval of the institutional ethics committee.

### Sample Size Estimation

The sample size was calculated using G*Power software version 3.1.9.7. The determination of sample size was based on a power of 80% and a significance level of 5% using the one-way analysis of variance (ANOVA) for four age groups of participated subjects. According to the mean and standard deviation (SD) of the cVEMPs p13 latency for age groups of 30 years old–40 years old, 40 years old–50 years old, 50 years old–60 years old and 60 years old–70 years old (12). The minimum sample size required was four subjects for each age group for cVEMPs. For oVEMPs testing, the sample size calculation was based on the oVEMPs reponse, i.e. the latency and amplitude of the n10 peak mean and SD of subjects aged less than 50 years old, 50 years old–59 years old and 60 years old–69 years old (11). Thus, the minimum sample size required for oVEMPs analysis was 12 subjects for each group.

### Testing Protocols

This study used testing protocols, stimulus recording techniques and parameters recommended by past studies (16, 22, 23). The 750 Hz TB acoustic stimuli (rise/fall time 0 ms, plateau 2.67 ms, stimulation rate 5/s) were used. Subjects were seated on a chair and the cVEMPs stimuli were delivered monaurally at 100 dBnHL with condensation polarity, using ER-3A insert earphones. oVEMPs were delivered using a hand-held Brüel and Kjaer (B&K) Mini-Shaker type 4810 (Naerum, Denmark) at 50 decibels normal Hearing Level (dBnHL). After careful calibration of the stimuli, these intensity levels were selected after ensuring that the levels were optimal in eliciting VEMPs reflexes (16, 24). Eclipse EP25 Evoked Potential System (Interacoustics, Denmark) was used to record both cVEMPs and oVEMPs.

### cVEMPs Testing Procedures

For cVEMPs testing, active electrodes were placed on the upper half of the left and right SCM muscles, a shared reference electrode was placed on the right upper sternum, and a ground electrode was placed on the high forehead. Before electrode placement, skin surfaces were scrubbed to ensure skin impedance of 5 kΩ or lower. The response window was set within 50 milliseconds (ms) and averaged over 200 stimuli for each run. The signal was band-pass filtered between 20 Hz and 2000 Hz. The ipsilateral recording was employed, where subjects were asked to turn their head opposite the contracted SCM muscle.

SCM muscle tension was monitored using an electromyography (EMG) level meter and also via acoustic feedback. The muscle tension was maintained between 100 μV and 150.6 μV to optimise cVEMPs recording. The EMG amplitude normalisation was performed using the EMG magnitude estimations obtained from the root mean square (RMS) of the EMG recording before stimulus onset.

### oVEMPs Testing Procedures

For oVEMPs, active electrodes were placed under the inferior eyelids in line with the pupils. A common reference electrode was placed below the right active electrode, while the ground electrode was placed on the chin. Subjects were asked to maintain their upward gaze (approximately 25° upward, by fixating at a point marked on the ceiling) and they were encouraged to maintain their gaze throughout the recording. Upward gaze ensures that the inferior oblique muscles’ belly was brought up beneath the skin for optimal recording (25).

The hand-held minishaker was placed on the subject’s high forehead (i.e., at the Fz site). The minishaker was held perpendicularly and a mark was made on the Fz site to maintain consistency throughout the stimulation. The recording was done within a 50 ms time window, with 80 stimuli averaged for each run. The EMG signal was amplified and band-passed filtered between 20 Hz and 500 Hz (22).

### Data Analysis

Interpretation of both cVEMPs and oVEMPs responses (including present/absent waveforms and peaks identification) were performed by both authors, who are experienced clinicians in the field. Present cVEMPs waveforms responses were identified at two distinct peaks, labelled as p13 and n23. These peaks latencies (in ms) and p13n23 peak-to-peak amplitude (in μV) were then analysed. The asymmetry ratio was calculated using the following formula:


Asymmetry ratio=(p13n23 max-p13n23 min/p13n23 max+p13n23 min)

For oVEMPs, two distinct peaks were also identified and labelled at approximately 10 ms and 15 ms (labelled as n10 and p15). The n10 and p15 latencies (in ms) and n10p15 peak-to-peak amplitude (in μV) were then analysed. The asymmetry ratio was calculated using the following formula:


Asymmetry ratio=(n10p15 max-n10p15 min/n10p15 max+n10p15 min)

Mean and SD were used for continuous data while frequencies and percentages were presented for categorical data. Statistical analyses were performed using IBM SPSS^®^ version 24.0 (SPSS Inc, Chicago IL). Shapiro-Wilk was performed to identify data normality. Statistical significance was interpreted when the *P*-value was less than 0.05. cVEMPs and oVEMPs responses for both right and left ears were pooled for analysis. An independent *t*-test was performed to identify the differences between gender for both cVEMPs and oVEMPs parameters. The ANOVA between groups was used to identify the effects of age groups for both cVEMPs and oVEMPs parameters (latencies, amplitudes and asymmetry ratios) among subjects who exhibited at least a unilateral response. Person’s chi-squared test was performed to determine any possible differences in the cVEMPs and oVEMPs responses for subjects aged 49 years old and below, and 50 years old and above.

## Results

Fifty healthy adults aged between 23 years old and 76 years old with a mean age of 51.56 (SD = 16.44) years old participated in this study. Nineteen (38%) were males and 31 (62%) were females. They were divided into four age groups: i) 23 years old–39 years old; ii) 40 years old–49 years old; iii) 50 years old–59 years old and iv) 60 years old and above. Ten (20%) subjects were recruited in each of the first three age groups and 20 (40%) subjects in the fourth age group.

cVEMPs were bilaterally absent in five (10%) subjects and unilaterally absent in 16 (32%) subjects (*n* = 26 ears [26%]). The response rates for subjects aged between 23 years old–39 years old and 40 years old–49 years old were 100% and 90%, respectively. However, the response rates reduced tremendously to 65% for subjects aged 50 years old–59 years old and 62.5% for subjects aged 60 years old and above. The Pearson’s chi-squared test found significant differences between the response rates for subjects aged 49 years old and below, and 50 years old and above (*χ*^2^ [1, *N* = 100] = 13.19, *P* < 0.001).

**Figure 1:**
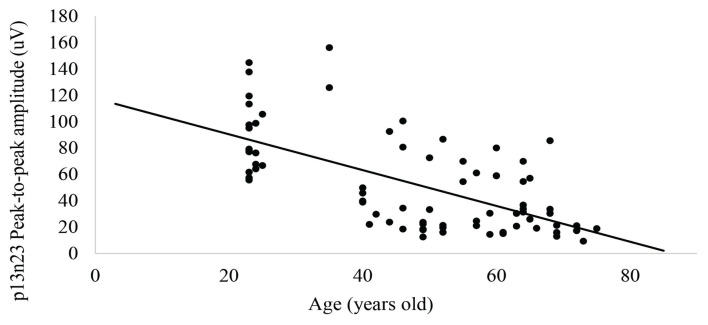
A linear regression curve fit on the age (in years old) effects on cVEMPs p13n23 peak-to-peak amplitude

**Figure 2:**
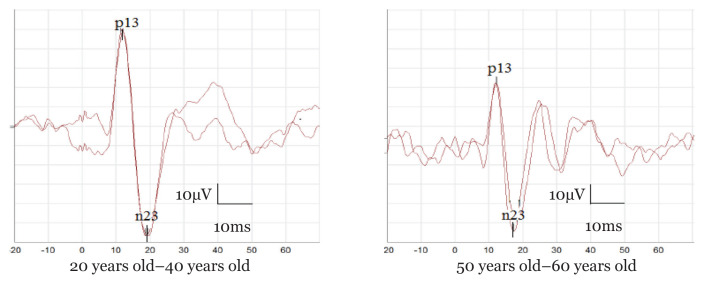
An example of a larger cVEMPs peak-to-peak amplitude of a subject aged 20 years old–40 years old (left) compared to a subject aged between 50 years old and 60 years old (right)

Mean and SD of cVEMPs latencies, p13n23 peak-to-peak amplitudes and asymmetry ratio among subjects who exhibited at least one cVEMPs response (*n* = 76 ears [76%]) were shown in Table 1. ANOVA revealed no significant differences between age groups for both p13 and n23 latencies or asymmetry ratio (Table 1). However, there was a significant difference between age groups for p13n23 peak-to-peak amplitude (*P* < 0.001). Tukey’s post-hoc analysis revealed significant differences in the cVEMPs amplitudes between subjects aged 23 years old–39 years old and 40 years old–49-year-old (*P* < 0.001), and between subjects aged 50 years old–59 years old and 60 years old and above (*P* < 0.001). There were no significant differences in the cVEMPs amplitudes between subjects aged 40 years old–49 years old and 50 years old–59 years old (*P* = 0.996), 40 years old–49 years old and 60 years old and above (*P* = 0.977), and between 50 years old–59 years old and 60 years old and above (*P* = 0.929). Using Pearson’s correlation coefficient, the analysis revealed a significant moderate negative correlation between age and amplitude (*r* = −0.593, *P* < 0.001). Figure 1 shows the scatter plot for p13n23 peak-to-peak amplitude as a function of age among subjects who elicited cVEMPs responses. Examples of cVEMPs response traces in two different age groups were shown in Figure 2.

There were also no significant differences between males and females subjects for latencies, amplitude and asymmetry ratio (*P* > 0.091) (Table 1). Overall, the mean latencies for this group of subjects were 14.02 (SD = 2.18) ms for p13 and 22.00 (SD = 2.06) ms for n23. The mean for p13n23 peak-to-peak amplitude was 52.23 (SD = 35.88) μV and asymmetry ratio was 0.2 (SD = 0.16).

The same subjects who were tested for cVEMPs also underwent oVEMPs testing. oVEMPs were absent in 13 (26%) subjects (nine unilateral and four bilateral). The total response rate for oVEMPs was *n* = 83 ears (83%). Similar to cVEMPs, the oVEMPs response rates also declined with age. The response rates for subjects aged 23 years old–39 years old and 40 years old–49 years old were 100% and 95%, respectively. The response rates reduced drastically at the beginning of the sixth decade of life; 75% response rates for 50 years old–59 years old and 72.5% for the age group of 60 years old and above. There was a significant difference in the proportion of oVEMPs absent responses between the age groups of 49 years old and below and 50 years old and above (*χ*^2^[1, *N* = 100] = 9.93, *P* = 0.002).

**Figure 3:**
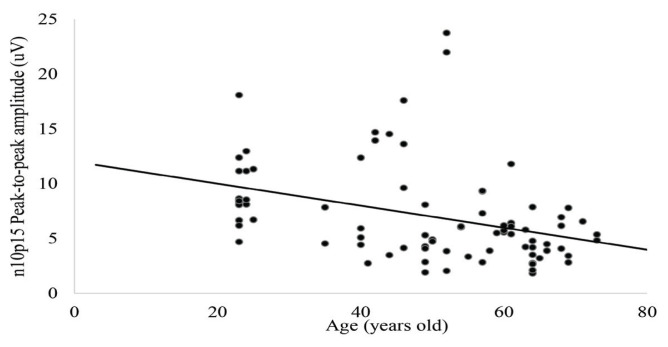
A linear regression curve fit on the age (in years old) effects on oVEMPs n10p15 peak-to-peak amplitude

**Figure 4:**
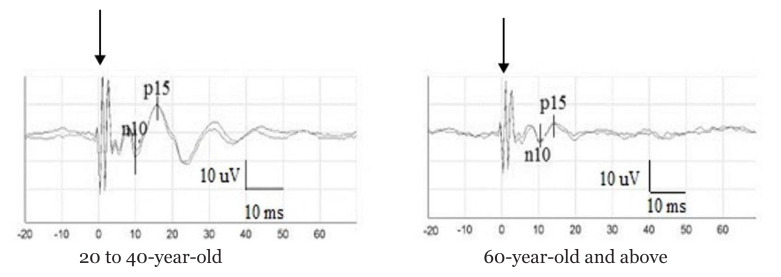
An example of a larger oVEMPs peak-to-peak amplitude in a subject, aged 20 years old–40 years old (left), compared to a subject aged 60 years old and above (right). Stimulus artifact is present at 0 ms, as indicated by the arrows

The mean (SD) for n10 and p15 latencies, n10p15 peak-to-peak amplitude and asymmetry ratio among subjects who elicited at least unilateral oVEMPs responses (*n* = 83 ears) were presented in Table 2. ANOVA revealed no significant differences for n10 and p15 latencies or asymmetry ratio in different age groups (*P* > 0.097) (Table 2). However, there was a significant difference between age groups for the n10p15 peak-to-peak amplitude (*P* = 0.01). Tukey’s post-hoc test revealed that the oVEMPs peak-to-peak amplitude was significantly lower for subjects aged 60 years old and above compared to the 23 years old–39 years old group (*P* = 0.008). However, there were no significant differences in oVEMPs peak-to-peak amplitude between the age groups of 23 years old–39 years old and 40 years old–49 years old (*P* = 0.804) or 50 years old–59 years old (*P* = 0.771). Pearson correlation revealed a significant moderate negative correlation between age and peak-to-peak amplitude (*r* = –0.466; *P* < 0.001). The effect is demonstrated as a scatter plot of n10p15 peak-to-peak amplitude as a function of age among subjects who elicited oVEMPs responses (Figure 3). Examples of oVEMPs traces in different age groups were displayed in Figure 4.

There were also no significant differences between males and females for oVEMPs latencies, peak-to-peak amplitude or asymmetry ratio (Table 2). Overall, the mean oVEMPs latencies in this group of subjects were 10.60 (SD = 1.19) ms for n10 and 15.62 (SD = 1.68) ms for p15. The mean asymmetry ratio for this cohort was 0.19 (SD = 0.15).

## Discussion

This study aims to identify changes in the cVEMPs and oVEMPs responses in different age groups of healthy adults. Absent cVEMPs responses increased with age, especially for subjects aged 50 years old and above. We reported a 95% cVEMPs response rate for subjects aged below 50 years old. However, the number reduced to 65% for the 50 years old–59 years old and 62.5% for 60 years old and above. This finding is in line with previous reports that a decline in cVEMPs response rate is notable with age increment (11, 12). Earlier research also reported that only 31% of subjects aged 65 years old and above elicited cVEMPs responses (26).

There were no significant changes in the p13 and n23 latencies with increasing age, using the 750 Hz TB stimuli. Previous studies also reported that ageing did not change p13 latency (14, 26, 27) or n23 latency (13, 14). However, some studies reported delayed latencies with increasing age for p13 latencies (12, 13) and n23 latencies (12). The delayed latencies could be due to testing parameters used in the different studies or possibly due to age-related physiological changes.

We also found that the cVEMPs peak-to-peak amplitude decreased by the age of 40 years old. Most studies also found that the p13n23 peak-to-peak amplitude decreased with increasing age, indicating that age-generated changes affect the otolithic organs (11–13, 26–29). There is broad published literature on the decreased physiological function of the vestibular system due to ageing, from the peripheral tissues up to the brainstem level. Ageing can lead to decreased function in sensory hair cells (30), Scarpa’s ganglion, vestibular nerve fibres (31, 32) and also vestibular nucleus in the brainstem (33).

A study reported that the cVEMPs amplitude decreased with the reduction of EMG level measured from SCM muscles contraction, in keeping with increasing age (28). Subjects with EMG levels less than 35 μV were excluded from this study to reduce the EMG level effect on the cVEMPs response. Nevertheless, this reduction in amplitude might not be directly attributable to the EMG level (28). A study reported that among the elderly subjects, 31% had absent cVEMPs responses, despite good SCM muscle contraction (26). Absent cVEMPs responses in the elderly indicates that age-related changes could occur within vestibular end organs or central vestibular pathways. Many studies found that the asymmetry ratio is more stable with increasing age than cVEMPs peak-to-peak amplitude. This is because the asymmetry ratio values represent the normalised responses of the interaural SCM muscles (11, 12, 14, 26). The findings indicate that otolith function and age-degenerative changes could occur in symmetry in all age groups. As the asymmetry ratio is more stable with age than the amplitude, the asymmetry ratio can be used to determine the otolith organs abnormalities especially for the elderly.

Similar results could be observed with the oVEMPs responses. There was a decreased in the oVEMPs response rate with increasing age, specifically at 50 years old and above. Response rates for oVEMPs decreased significantly to 75% and 74% in the 50s and above 60s. Our findings are consistent with a previous finding that reported only 77% of subjects aged 50 years old and above exhibited oVEMPs responses (34). Another study reported that half of the healthy individuals aged 40 years old and above had absent oVEMPs (17). However, we found no significant differences between age groups in the n10 and p15 latencies and asymmetry ratio among subjects who exhibited oVEMPs responses. A previous study also reported no significant changes in the oVEMPs latencies with increasing age (14). We found a significant reduction in the amplitude, especially for the 60 years old and above group. A study also reported a decrease in the amplitude with increasing age among healthy adult subjects (27), whereas another study found that amplitude decreased by 2.9 μV for every decade of age (11). Like cVEMPs, asymmetry ratio is more stable with increasing age than the amplitude measures (11).

The results of this study are consistent with previous recent studies, indicating age-degenerated changes in the otolith functions (11, 12, 14, 26–29). Table 3 shows the comparison of cVEMPs and oVEMPs responses with increasing age in different studies. Differences in VEMPs responses between this study and other studies could be due to the differences in testing parameters. Most studies used 500 Hz TB frequency (11–14, 26–29), while this study used 750 Hz TB stimuli, as the tuning curve of older subjects occurred at this frequency (18). However, the finding is consistent with previous studies that found reduced amplitude with age (11–14, 26–29), specifically after 40 years old for cVEMPs and 60 years old and above for oVEMPs. Besides testing parameters, other factors such as anatomical differences, ethnicity (11) and muscle bulk (35) might change VEMPs test findings. With the use of 750 Hz TB stimuli, we found no significant changes in latencies, while there was a significant amplitude reduction with increasing age. There were no significant differences between gender for latencies, peak-to-peak amplitude and asymmetry ratio for cVEMPs and oVEMPs.

This study did not include subjects aged 80 years old and above. Therefore effects of age on VEMPs responses were not identified for this age group. It is recommended for future studies to include a larger sample size from different age groups and Malaysian ethnicities. Further research on the use of bone conduction stimulus with higher intensity stimulation is also recommended, as the stimulus can elicit cVEMPs in cases where the responses are bilaterally absent, especially in the elderly (17).

## Conclusion

The cVEMPs and oVEMPs response rates decreased with increasing age, particularly after the age of 50 years old. As compared to peak-to-peak amplitude, latencies and asymmetry ratio were more stable with age in both cVEMPs and oVEMPs responses. These findings should alert clinicians on the possibility of absent cVEMPs and oVEMPs responses when used on older adults aged 50 years old and above. Latency and asymmetry ratio values are suitable to be used as a guide when VEMPs are performed in the elderly with any underlying peripheral vestibular disorders. This is because the need for age-corrective values for these parameters is reduced when using 750 TB stimuli.

To the best of our knowledge, this is the first published normative data for cVEMPs and oVEMPS in Malaysia and also Southeast Asia, obtained in healthy adults aged between 23 years old and 76 years old. Findings from this study could be used by health professionals in the region in diagnosing and managing vestibular related cases in their clinical settings.

## Figures and Tables

**Table 1:** Comparison of cVEMPs p13 and n23 latencies, p13n23 peak-to-peak amplitude and asymmetry ratio in different age groups and gender

Group	p13 latency mean (SD)	Statistic, *P-*value	n23 latency mean (SD)	Statistic, *P-*value	p13n23 peak-to-peak-amplitude mean (SD)	Statistic, *P-*value	Asymmetry ratio mean (SD)	Statistic, *P-*value
Age (years old)								
20–39	13.44 (2.32)	*F*(3,72) = 1.823; *P* = 0.151^a^	21.91 (2.17)	*F*(3,72) = 2.576; *P* = 0.060^a^	93.95 (30.61)	*F*(3,72) = 23.244; *P* < 0.001^a^*	0.11 (0.06)	*F*(3,27) = 2.762; *P* = 0.061^a^
40–49	4.99 (2.56)		23.10 (2.10)		38.32 (26.69)		0.20 (0.17)	
50–59	14.11 (1.56)		21.34 (1.90)		40.45 (25.01)		0.34 (0.21)	
≥ 60	13.75 (1.93)		21.64 (1.81)		35.01 (22.02)		0.22 (0.15)	
Gender								
Male	14.2 (2.24)	*t*(74) = 0.503; *P* = 0.616^b^	21.93 (2.22)	*t*(74) = −0.214; *P* = 0.831^b^	47.96 (36.37)	*t*(74) = −0.747; *P* = 0.457 b	0.28 (0.19)	*t*(13.117) = 1.821; *P* = 0.091^b^
Female	13.93 (2.17)		22.04 (1.99)		54.46 (35.79)		0.16 (0.13)	

Notes:

a One-way analysis of variance (ANOVA);

b Independent *t*-test;

* Significant at *P* < 0.05

**Table 2:** Comparison of oVEMPs n10 and p15 latencies, n10p15 peak-to-peak amplitude and asymmetry ratio in different age groups and gender

Group	n10 latency mean (SD)	*P-*value	p15 latency mean (SD)	*P-*value	n10p15 peak-to-peak-amplitude mean (SD)	*P-*value	Asymmetry ratio mean (SD)	*P-*value
Age (years old)								
20–39	10.32 (0.89)	*F*(3, 79) = 2.177; *P* = 0.097^a^	15.54 (1.38)	*F*(3,79) = 1.742; *P* = 0.165^a^	9.02 (3.13)	*F*(3,79) = 4.071; *P* = 0.010^a^*	0.18 (0.10)	*F*(3, 33) = 0.692; *P* = 0.564^a^
40–49	10.20 (0.57)		14.93 (1.66)		7.82 (5.03)		0.25 (0.19)	
50–59	11.00 (1.63)		16.08 (1.87)		7.66 (6.54)		0.14 (0.19)	
≥ 60	10.85 (1.32)		15.89 (1.72)		5.05 (4.38)		0.19 (0.14)	
Gender								
Male	10.80 (1.43)	*t*(81) = 1.193;	15.38 (1.86)	*t*(81) = −1.018;	6.06 (2.92)	*t*(80.671) = −1.967; *P* = 0.053^b^	0.22 (0.19)	*t*(19.033) = 0.854; *P* = 0.403^b^
Female	10.48 (1.0)	*P* = 0.236^b^	15.77 (1.56)	*P* = 0.312^b^	7.77 (5.00)		0.17 (0.12)	

Notes:

a One-way analysis of variance (ANOVA);

b Independent *t*-test;

* Significant at *P* < 0.05

**Table 3:** Comparison of the age range (years old), stimuli used and findings from previous studies and this study for both cVEMPs and oVEMPs

Studies	Age range (years old)	Stimuli	Findings
Li et al. (11)	26–92	500 Hz TB cVEMPs	Reduced amplitude with increasing age Reduced amplitude by 2.9 uV/decade
		Head tap oVEMPs	Prolonged n10 latency by 0.12 ms/decade
Singh et al. (12)	10–85	500 Hz TB cVEMPs	Reduced amplitude with increasing age Prolonged p13 and n23 latencies with increasing age
Maleki et al. (13)	19–79	500 Hz TB cVEMPs	Reduced amplitude with increasing age Prolonged p13 and n23 latencies with increasing age
Nguyen et al. (14)	20–70	Clicks, 500 Hz TB and head taps cVEMPs	Reduced amplitude with increasing age
Nguyen et al. (14)	20–70	Clicks, 500 Hz TB and head taps oVEMPs	Reduced amplitude with increasing age Prolonged n10 latencies with increasing age
Maes et al. (26)	18–80	500 Hz TB cVEMPs	Reduced amplitude with increasing age
Layman et al. (27)	26–98	500 Hz TB cVEMPs	Reduced amplitude with increasing age
		Head tap oVEMPs	Reduced amplitude with increasing age
Akin et al. (28)	22–86	500 Hz TB cVEMPs	Reduced amplitude with increasing age
Agrawal et al. (29)	70–93	500 Hz TB cVEMPs	Reduced amplitude with increasing age
		Head tap oVEMPs	Reduced amplitude with increasing age
This study	23–75	750 Hz TB cVEMPs	Reduced amplitude with increasing age
		750 Hz TB head tap oVEMPs	Reduced amplitude with increasing age

Notes: TB = tone burst; cVEMPs = cervical vestibular evoked myogenic potentials; oVEMPs = ocular vestibular evoked myogenic potentials
